# Heterogeneous lineage marker expression in naive embryonic stem cells is mostly due to spontaneous differentiation

**DOI:** 10.1038/srep13339

**Published:** 2015-08-21

**Authors:** Gautham Nair, Elsa Abranches, Ana M. V. Guedes, Domingos Henrique, Arjun Raj

**Affiliations:** 1 Department of Bioengineering, University of Pennsylvania, Philadelphia, PA 19104, USA; 2 Instituto de Medicina Molecular and Instituto de Histologia e Biologia do Desenvolvimento, Faculdade de Medicina da Universidade de Lisboa, Lisboa, Portugal; 3 Champalimaud Neuroscience Programme, Champalimaud Centre for the Unknown, Doca de Pedroucos, Lisboa, Portugal

## Abstract

Populations of cultured mouse embryonic stem cells (ESCs) exhibit a subfraction of cells expressing uncharacteristically low levels of pluripotency markers such as Nanog. Yet, the extent to which individual Nanog-negative cells are differentiated, both from ESCs and from each other, remains unclear. Here, we show the transcriptome of Nanog-negative cells exhibits expression of classes of genes associated with differentiation that are not yet active in cells exposed to differentiation conditions for one day. Long non-coding RNAs, however, exhibit more changes in expression in the one-day-differentiated cells than in Nanog-negative cells. These results are consistent with the concept that Nanog-negative cells may contain subpopulations of both lineage-primed and differentiated cells. Single cell analysis showed that Nanog-negative cells display substantial and coherent heterogeneity in lineage marker expression in progressively nested subsets of cells exhibiting low levels of *Nanog*, then low levels of *Oct4*, and then a set of lineage markers, which express intensely in a small subset of these more differentiated cells. Our results suggest that the observed enrichment of lineage-specific marker gene expression in Nanog-negative cells is associated with spontaneous differentiation of a subset of these cells rather than the more random expression that may be associated with reversible lineage priming.

Mouse embryonic stem cells (ESCs) are self-renewing, pluripotent cells derived from the inner cell mass of mouse blastocysts, the portion of early embryos that gives rise to the embryo proper. Given appropriate cues, ESCs can differentiate into a variety of cell types. However, this directed differentiation is often incomplete in that some cells do not respond appropriately, suggesting heterogeneity within cultured populations of stem cells. Indeed, even while maintained in conditions explicitly formulated to promote pluripotency, such as those containing inhibitors of FGF/ERK and GSK3 signaling^1^, a small fraction of ESCs will spontaneously exhibit low levels of expression of transcription factors associated with pluripotency^2^,^3^,^4^. In live cells, variability has been observed for reporters of *Stella* (*Dppa3*), *Rex1* (*Zfp42*) and *Nanog* expression^5^,^6^,^7^,^8^,^9^,^10^, and recently published data suggests that NANOG fluctuations are widespread in ESCs, even in the presence of inhibitors of FGF/ERK and GSK3 signaling, allowing ESCs to explore different lineage options^11^.

These pluripotency factor-negative cells make up a stable fraction of the total population^12^. Stem cell cultures often contain cells that have spontaneously differentiated and could account for some of the pluripotency factor(−) cells. Alternatively, these cells could be transiently exploring a state that is predisposed to differentiation, but may still return to pluripotency, a phenomenon known as “lineage priming”^10^,^13^,^14^. Technical difficulties associated with reporter gene infidelity have made it difficult to definitively distinguish between spontaneous differentiation and reversible lineage priming. In particular, while many of these studies have examined the differences in gene expression in the low-pluripotency-marker-expression subpopulation and shown that these cells exhibit widespread transcriptome differences, they have not applied single cell analysis to this subpopulation. As such, the exact nature of these cells remains unclear, in particular, whether the lineage markers often found in these cells are expressed in sporadic, transient fluctuations, or whether they are instead expressed in a manner consistent with a coherent departure from pluripotency akin to that of natural differentiation processes. The latter would be surprising given that “2i” growth conditions in which FGF/ERK and GSK3 signaling are inhibited are thought to strongly inhibit spontaneous differentiation^1^. Still, the existence of cell-to-cell heterogeneity in NANOG levels in 2i growth conditions leaves open the question of whether low-NANOG ESCs are undergoing lineage priming or escaping inhibition to enter spontaneous differentiation.

In this paper, we used RNA-sequencing and single cell transcript counting to characterize the nature of ESCs with low levels of *Nanog*. Using an ESC line containing a rapidly-responding fluorescent *Nanog* reporter^12^ to isolate low and high Nanog cells, we measured the expression of a variety of lineage and developmental genes in these subpopulations, and identified a subset of these with increased expression in Low Nanog cells. Transcription factor binding site analysis suggested that the expression of these genes is not under direct control of the pluripotency network, indicating the presence of spontaneously differentiated cells. We also compared the transcriptome signature of ESC grown in 2i conditions to that of ESCs allowed to enter differentiation for a day by removing pluripotency signaling factors from the growth media. We found that this differentiation resulted in relatively small changes in the expression of the same lineage and developmental genes, suggesting that the stochastically arising population of cells with low levels of Nanog include cells that have deviated relatively far from pluripotency. Using single cell RNA FISH^15^ to count transcripts in thousands of individual cells, we found that the lineage marker expression was not the result of random transient fluctuations in gene expression, but rather due to concerted changes in expression in single cells. Our analysis show that two-thirds of the cells with low Nanog mRNA display a deeply entrenched loss of pluripotency, with a subpopulation of them exhibiting surprisingly well-developed lineage differentiation programs. Our data supports a hierarchical model in which progressive subpopulations exhibit increasing levels of deviation from pluripotency. Our results suggest that the occurrence of lineage marker expression in Nanog-negative cells might not only be associated with reversible lineage priming but also, and perhaps primarily, with spontaneous differentiation events.

## Results

### Expression of pluripotency and lineage markers in heterogeneous and differentiated ESCs

We first wanted to compare the degree to which cells grown in pluripotency conditions (2i + LIF) while exhibiting low NANOG levels differ in their transcriptome signature from cells exhibiting high NANOG levels. We also compared those changes to the transcriptome changes that transpire when the entire population is subjected to differentiation conditions (N2B27) for a short period of time (one day).

**Figure 1:**
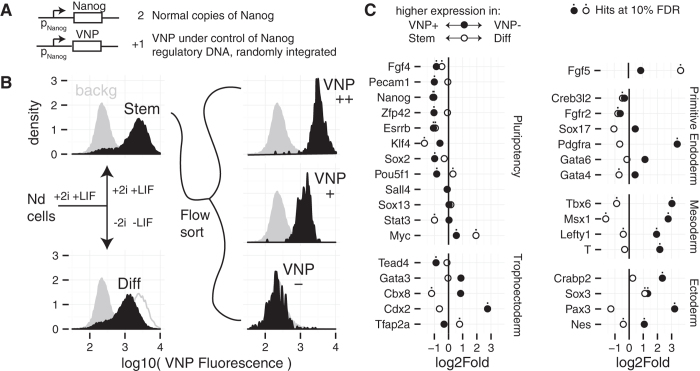
Experiment Diagram and Differential Expression of Selected Genes. (**A**) Simplified diagram of the construction of the Nd Nanog reporter cell line^12^. In the Nd cell line Venus (VNP) fluorescence is a reporter of Nanog expression. (**B**) Diagram and Nanog:VNP flow cytometry profiles of the samples from which RNA was extracted for RNA-Seq. The solid grey profile in all panels corresponds to fluorescence cytometry of the parent E14tg2a cell line used as a background control. In the Diff panel, the Stem data is shown with a grey outline for comparison. (**C**) RNA expression fold changes between the Nanog:VNP(+) and Nanog:VNP(−) (filled circles) samples and between the Stem and Diff (open circles) samples for several genes involved in pluripotency or in early extraembronic and embryonic lineages. Negative points denote genes whose expression is higher in Nanog:VNP(+) or Stem samples, while positive points denote genes whose expression is higher in Nanog:VNP(−) or Diff samples. Dots mark genes that also turn up as hits for significant differential expression at a 10% false discovery rate.

To accomplish this, we used the Nd reporter ESC line^11^,^12^, which contains a short-lived fluorescent reporter (Venus-NLS-PEST, VNP) under the control of the Nanog genomic region (Fig. 1A). We sorted cells grown for 48 hours in medium containing FGF/ERK and GSK3 inhibitors (2i) and LIF (ground-state conditions) based on levels of reporter expression into both high (Nanog:VNP(+)) and low (Nanog:VNP(−)) populations (Fig. 1B). We also isolated a subpopulation that expressed VNP at higher levels (Nanog:VNP(++)), but found its population-level transcriptome to be similar to that of Nanog:VNP(+), and so did not consider it further (Supplementary Fig. S1). In parallel, we removed 2i and LIF from the unsorted culture for 24 hours to study early events of culture-wide differentiation (N2B27 media) (Fig. 1B). We isolated RNA from each of these populations and performed RNA-seq to compare expression levels between the various conditions, in particular: i) Nanog:VNP(+) to Nanog:VNP(−) and ii) culture-wide differentiation (denoted “Diff”) to ESCs maintained in 2i + LIF medium (denoted “Stem”). Note that in our experimental design, it is possible that transient effects due to the switch between culture conditions may confound some of our comparisons. However, additional analyses we describe below suggested that the transcriptome of the Nanog:VNP(−) cells we analyzed are similar in character to those grown for longer periods in 2i + LIF conditions.

We initially looked at the expression fold-changes for several known pluripotency markers and early lineage specification genes (Fig. 1C). We found that the expression of pluripotency markers was consistently reduced in the Nanog:VNP(−) cells relative to Nanog:VNP(+). These cells also showed strong up-regulation of various lineage markers. On the other hand, comparing Diff to Stem conditions revealed that the levels of the important markers Oct4 (Pou5f1), Rex1 (Zfp42), and Pecam1 were relatively unchanged, although some pluripotency markers were repressed in Diff conditions. Remarkably, 231/633 of the lineage markers up-regulated in the Nanog:VNP(−) cells, such as Tbx6, Msx1, and Nes, are down-regulated after a day of differentiation, a finding we address later. The Hox genes are also uniformly and strongly up-regulated in Nanog:VNP(−) cells but if anything down-regulated on culture-wide differentiation (Supplementary Fig. S2).

These initial comparisons suggest that the Nanog:VNP(−) cells that stochastically arise in ground state conditions exhibit signs of strong deviations from the pluripotent state. These deviations appear to be more extreme than for cells subjected to 24 hours of differentiation, thus making the relatively shallow departures thought to be associated solely with lineage priming seem unlikely and rather leading us to suggest that a considerable fraction of NANOG-negative cells must have spontaneously entered differentiation. One possible explanation for the appearance of these cells might be their incomplete transition to ground state conditions, given that they were analysed 48 hours after transfer from serum+LIF to 2i + LIF.

In order to verify that two days of culture was sufficient to reach equilibrium in 2i + LIF conditions, we compared their gene expression profiles with those we recently published using multiplex RT-qPCR for a panel of pluripotency and lineage-affiliated genes obtained from mESCs grown in 2i + LIF for 6 days (3 passages) (Supplementary Fig. S3)^11^. We observed a very strong correspondence in the fold-changes between Nanog:VNP(−) and Nanog:VNP(+) cells in the two datasets, suggesting that differences in gene expression are established as soon as after 2 days in 2i + LIF, and are similar to the ones observed after longer culturing in 2i + LIF. Furthermore, the single molecule RNA FISH distributions for *Nanog* and *Oct4* mRNAs also do not appreciably change when comparing 2 days to 6 days in 2i + LIF (Supplementary Fig. S4). These results demonstrate the existence of Nanog(−) cells even in long term 2i + LIF culture (as also documented in^16^). For these reasons, we favor the interpretation that our results are not a transient consequence of media transfer.

Together, these experiments suggest that the transcriptional profile of the Nanog:VNP(−) population isolated from cells grown in 2i + LIF for 2 days is similar to those grown for longer periods of time, although it is possible that other transient effects due to the shift in growth medium may affect the interpretation of our results.

### Genome-wide analysis of gene expression differences

**Figure 2:**
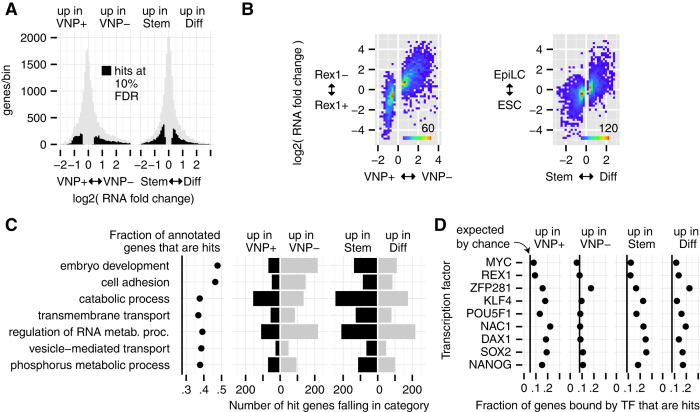
Genome Wide Analysis of Heterogeneity and Differentiation. (**A**) Distribution of fold changes for all genes in our study between Nanog:VNP(+) and Nanog:VNP(−) subpopulations (left) and between Stem and Diff conditions (right). Black regions indicate genes that are significant hits at a 10% false discovery rate. (**B**) Joint distribution of fold changes for genes between this work and literature data. Left 2 panels include only genes that are hits in Nanog:VNP(+)/Nanog:VNP(−) and compare Nanog:VNP(+)/Nanog:VNP(−) fold change to reported RNA-Seq fold changes for *Rex1*+/*Rex1*- populations grown i serum + LIF^8^. Right panel: Includes only genes that are hits in Stem/Diff and compares to reported microarray fold changes between ESCs and day1 EpiLCs^6^. (**C**) The set of GO categories selected by our “greedy” method for genes that are hits in either Nanog:VNP(+)/Nanog:VNP(−) or Stem/Diff, using a minimal GO size of 750 genes, in the order that they were selected (most relevant first). Leftmost panel: Fraction of genes in GO category that are a hit, with the vertical line showing the (background) fraction of all genes that are hits. Right 4 panels: Within each panel, each hit gene is assigned to the topmost category that it is annotated for. (**D**) For each transcription factor, number of genes called as bound in literature^20^ that are also hits of the specified type, divided by the number of bound genes. Black vertical lines indicate the fraction of hits in our gene universe.

Owing to the depth of our sequencing and high degree of fidelity between replicates (Supplementary Fig. S5), we were able to characterize differences in the transcriptomes at a high level of precision. Nearly half the genes with fold changes of at least 40% in Nanog:VNP(+) vs Nanog:VNP(−) were called as differential expression hits at a false discovery rate (FDR) of 10% (Fig. 2A). Of these 3293 hits, 1993 are more highly expressed in Nanog:VNP(−) than Nanog:VNP(+) and 1300 are more highly expressed in Nanog:VNP(+). In our differentiation comparison, we calculated 4233 gene hits differentially expressed between the 2i + LIF population (Stem) and after 24 h without 2i and LIF (Diff) (Fig. 2A). Due to even higher replicate reproducibility, these hits include genes with RNA fold changes as small as 20%. In this case, 2235 gene hits were down-regulated and 1998 up-regulated after a day of differentiation. Our Nanog:VNP(+/−) results correlate closely with RNA-seq-based expression differences between Rex1(+) and (−) cells^8^, and our Stem/Diff results are in concordance with earlier findings which compare ESCs and day 1 EpiLCs in differentiation conditions^6^ (Fig. 2B). Interestingly, we found that the expression changes we observed in Nanog:VNP(−) cells did not correlate with those previously observed after Nanog depletion (Supplementary Fig. S6)^17^, suggesting that the altered transcriptome of Nanog:VNP(−) cells does not reflect functional effects of Nanog levels *per se*.

We then analyzed the molecular function of the sets of differentially expressed genes in Nanog:VNP(−) cells obtained under pluripotency conditions (2i + LIF) and directed differentiation (N2B27 alone). The standard means of doing such an analysis is to use gene ontology analysis; however, such analyses tend to produce a list of redundant, overlapping functional terms. To produce a more comprehensible list, we developed a “greedy” method to describe a set of genes in terms of gene ontology categories. In short, we prioritize categories that are highly concentrated for genes of interest, but without letting multiple categories claim priority based on the same gene (see Experimental Procedures for details). Applying this algorithm to our data, we found (Fig. 2C) that embryo development is the most important molecular function of the differentially expressed genes in our study (including both the Nanog:VNP(+)/Nanog:VNP(−) and Stem/Diff comparisons), closely followed by cell adhesion, a function that in earlier studies has been associated with early developmental changes. Also notice in (Fig. 2C) that the set of genes up-regulated in Nanog:VNP(−) cells is more heavily weighted towards embryo development than the set of genes up-regulated upon culture-wide directed differentiation. We also carried out a more conventional GO analysis using goseq^18^, which confirmed the overall nature of the categories revealed by Greedy GO, although with less discrimination between some categories (Supplementary Table S3).

We argue that embryo development and lineage marker gene expression is the most salient feature of the transcriptional profile of cells with low levels of Nanog found in pluripotency conditions. As shown in Supplementary Fig. S7, the lineage-associated genes from Fig. 1C are found towards the extremes of the fold change-expression level distribution of our hits, and half of the genes with comparably extreme changes are also annotated for roles in development and pattern specification.

### Coherent vs. incoherent expression of Nanog:VNP(−) vs. Diff

**Figure 3:**
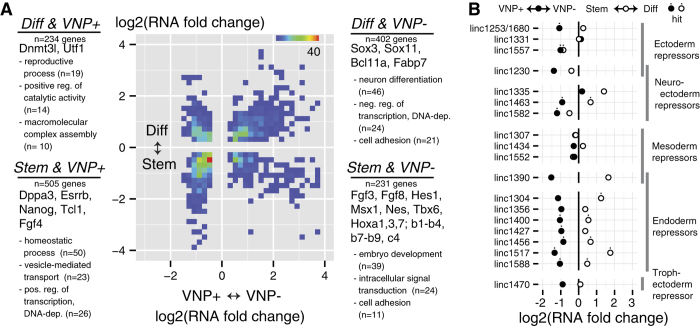
Distribution of Joint Hits and Differential Expression of lincRNA. (**A**) Joint distribution of fold changes for genes that turned up as 10% FDR hits for differential expression in heterogeneity (Nanog:VNP(+) vs Nanog:VNP(−)) and differentiation (Stem vs Diff). Text annotations for each quadrant note some genes found in it, as well as the top 3 GO terms and number of genes falling in them by the “greedy” method. (**B**) Differential expression analysis for lincRNA identified by^22^ as repressors of certain lineage programs in mouse ES-cells.

To further dissect the differences between ESCs grown in 2i + LIF conditions and (1) cells grown in these conditions that exhibit low levels of Nanog expression vs. (2) cells subjected to a short period of differentiation, we compared the fold changes for genes that turned up as 10% FDR hits in both comparisons (Fig. 3A). Of the 1372 differentially expressed genes, about two-thirds were altered in “coherent” directions (genes both up- or down-regulated in Nanog:VNP(+)/VNP(−) and Stem/Diff - lower-left and upper-right quadrants), while the remaining one third are differentially expressed in “incoherent” directions.

Genes that are repressed in both comparisons (the lower-left Stem and Nanog:VNP(+) quadrant in Fig. 3A) can be interpreted as being part of the pluripotency network, and, as expected, this group includes known pluripotency regulators such as Nanog, Esrrb, and Stella (Dppa3). The top-right Diff and Nanog:VNP(−) quadrant in Fig. 3A can be interpreted to include genes that form part of a “common core” between the processes of spontaneous differentiation and short-term culture-wide differentiation. Assuming that 24 hours exposure to differentiation conditions is sufficient to trigger definitive differentiation along several paths (mesoderm, endoderm and neuroectoderm), this group should be enriched in early differentiation-associated genes. Indeed, applying the “greedy” gene ontology method used before, we found that neural differentiation is the most important molecular function represented by the genes in this quadrant, which is reasonable given that the medium we used (N2B27 medium) is known to favor neural lineages^19^. Nonetheless, markers characteristic of other germ layers were also present, suggesting that these cells have retained some aspects of pluripotency.

The remaining top-left and bottom-right “incoherent” quadrants include 459 genes in total. The genes up-regulated in Nanog:VNP(−) but down-regulated on culture-wide directed differentiation include many lineage markers, and the Hox genes. Molecular function analysis shows that embryonic development is indeed the most prominent feature of this quadrant. What is interesting is that these genes are also significantly repressed upon culture-wide directed differentiation. One way to rationalize this phenomenon starts by supposing that ESC cultures have low levels of expression of lineage markers and developmental genes for multiple pathways. This could be either because of the existence of spontaneously differentiated cells in the Nanog:VNP(−) population, or because of lineage marker expression in still-pluripotent cells (our RNA FISH results described later argue for the former possibility). Then, upon culture-wide differentiation, all but a few lineage pathways may be shut down, as indicated by the apparent bias towards neural lineages observed after culture-wide differentiation. We thus propose that this bottom-right quadrant is composed mostly of non-neural lineage genes that are up-regulated during spontaneous differentiation but moderately down-regulated after 24 hours in culture-wide differentiation conditions.

Molecular function analysis of the 234 genes contained in the top-left quadrant, showing up-regulation both in pluripotent Nanog:VNP(+) cells and upon culture-wide differentiation, does not suggest any single function that defines this class, and manual inspection turned up few genes, aside from Utf1 and Dnmt3l, that have well-known roles in development.

### Pluripotency TF binding analysis of Nanog:VNP(+) vs. Nanog:VNP(−) and Stem vs. Diff

The large number of differentially expressed genes in our analysis led us to ask how many were direct targets of the pluripotency transcriptional network. We used published ChIP-seq data^20^ for pluripotency transcription factors in mouse ESCs to determine which genes the pluripotency factors bind to, and then calculated what percentage of these genes were differentially expressed when comparing either Nanog:VNP(+) to Nanog:VNP(−) or Stem to Diff (Fig. 2D).

We found that pluripotency transcription factor binding is associated with a gene being up- or down-regulated upon culture-wide differentiation (Diff and Stem columns in Fig. 2D) and for a genes with high expression in pluripotent cells (Nanog:VNP(+) column), but binding does not provide any information as to which genes are up-regulated in Nanog:VNP(−) cells. The former is to be expected given that pluripotency transcription factors are known to act in part through positive regulation of each other, and that they bind many genes that are repressed in the pluripotent state and up-regulated upon differentiation^20^,^21^. It was surprising, however, to find that genes upregulated in Nanog:VNP(−) seem not to be, in general, under the control of the pluripotency network. Although some early lineage-specification genes, like the ones shown in Fig. 1C, are highly up-regulated in Nanog:VNP(−) and are bound by pluripotency transcription factors, these are the exception rather than the rule. This corroborates the finding that the transcriptome of Nanog:VNP(−) shows signatures of cellular states that have deviated far enough from pluripotency, so as to no longer be under the direct control of the pluripotency network, whereas cells in differentiation conditions for 24 hours are still controlled by the pluripotency network.

### Expression of long intergenic non-coding RNAs

Our RNA sequencing data also revealed changes in the expression of transcripts other than mRNAs, such as long intergenic non-coding RNA (lincRNAs). Since studies of lincRNAs expressed in ESCs indicate that they may be important regulators of pluripotency and embryonic differentiation^22^,^23^, we analyzed the differential expression of these specific RNAs in both pluripotency-associated heterogeneity and in directed differentiation conditions Fig. 3B and Supplementary Fig. S8). We observed that, out of 166 ESC-specific lincRNA^22^, 46% represented significant hits in one of our comparisons. Surprisingly, the ESC-specific lincRNAs are biased towards lower expression in Nanog:VNP(−) but higher expression in short-term differentiated cells (Fig. 3B). Thus, the lincRNAs are largely in the “incoherent” category of genes (Fig. 3A), for which we could not ascribe any particular molecular function. We also noticed that endoderm repressor lincRNAs had consistently lower expression in Nanog:VNP(−) and higher expression in short-term differentiated cells, suggesting that the Nanog:VNP(−) subpopulation might be permissive to endoderm specification.

### RNA FISH reveals that a considerable fraction of low Nanog cells have exited pluripotency

While RNA-seq provided us with a panoramic view of the gene expression profile in the Nanog:VNP(−) subpopulation, it does not provide any information about cell-to-cell variability within that subpopulation. We thus used single molecule RNA FISH to quantitatively measure expression levels of key genes within individual cells, with the use of multiple fluorophores enabling us to correlate the expression of up to 3 genes at a time.

**Figure 4:**
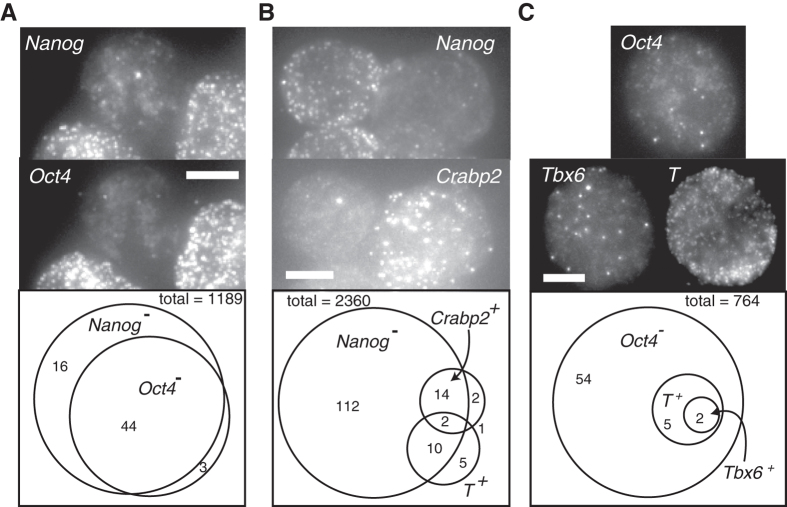
Single Cell Analysis of Transcriptome Heterogeneity. (**A**) Maximum projection images from Nd cells stained for *Nanog* and *Oct4* RNA. Scale bar in all panels is 5 um long. The *Nanog*(−)*Oct4*(−) cell in the center is flanked by *Nanog*(+)*Oct4*(+) cells. (bottom) Summary of RNA FISH results on 1189 single cells. The vast majority of cells are *Nanog*(+)*Oct4*(+). 60 cells are *Nanog*(−), and 44 of these are also *Oct4*(−). The (+/−) cutoffs were 30 and 80 transcripts for *Nanog* and *Oct4*, respectively (see Supplementary Fig. S9). (**B**) RNA FISH staining for *Nanog* and *Crabp2* (ectoderm marker) showing a *Nanog*(+)*Crabp2*(−) and a *Nanog*(−)*Crabp2*(+) cell. (bottom) Summary of simultaneous staining for *Crabp2*, *T* (*Brachyury*) and *Nanog*. The (+/−) cutoffs were 30 and 50 transcripts for *Crabp2* and *T* respectively (Supplementary Fig. S9). **(C)** RNA FISH staining for *Tbx6*, *T* and *Oct4*, showing a *Tbx6*(+) *T*(+) *Oct4*(−) cell. (bottom) Summary. The (+/−) cutoff for *Tbx6* was 10 transcripts (Supplementary Fig. S9).

First, to characterize the cells expressing low levels of Nanog mRNA, we co-hybridized cells with probes targeting *Nanog* mRNA together with probes targeting the pluripotency marker *Oct4*. By examining the single cell mRNA distributions for both Nanog and Oct4, we identified two clear subpopulations, which we denote *Nanog* mRNA (+/−) (cutoff of 30 mRNA molecules per cell) and *Oct4* mRNA (+/−) (cutoff of 80 mRNA molecules per cell) (Supplementary Fig. S9). As apparent from the distributions, this classification was not particularly sensitive to the particular threshold chosen because the distinction between the positive and negative populations was clear. Within the overall population, we found that around 5% (*N *= 1189) of cells had very low levels of *Nanog* mRNA (less than 30 transcripts per cell). We found that 70% of these *Nanog* mRNA(−) cells have very low *Oct4* mRNA levels (less than 80 transcripts per cell) (Fig. 4A, Supplementary Fig. S9). Since maintaining *Oct4* gene expression within a tight concentration interval is considered essential to the ESC state^24^, we inferred that these cells have likely exited pluripotency. In a separate experiment, we also found that the majority of *Nanog* mRNA(−) cells have also very low levels of Rex1 transcripts (Supplementary Fig. S10). It is also important to point out that the lack of mRNA does not necessarily imply a complete lack of protein expression. In the case of *Oct4*, the protein half-life is roughly 12 hours and the mRNA half-life is ~7.5 hours^25^. Thus, the relatively large fold change difference of mRNA suggests that the cell has not transcribed *Oct4* for some time, thus making it likely that the protein level has decreased significantly.

Note that the fold-changes in *Oct4* (4.5-fold reduction) and *Rex1* (8-fold) measured by RNA FISH in the *Nanog* mRNA(−) relative to the *Nanog* mRNA(+) cells was considerably larger than those we found by RNA-seq in the FACS-sorted Nanog:VNP(−) vs. Nanog:VNP(+) subpopulations (1.77 and 2.04-fold for Oct4 and Rex1, respectively). To explore this discrepancy, we analyzed RNA FISH data for both *Nanog* mRNA and our reporter Nanog:VNP mRNA. We found that approximately 50% of the Nanog:VNP mRNA (−) cells in 2i + LIF conditions are actually positive for *Nanog* mRNA and have the same distribution of *Nanog* mRNA levels as the Nanog:VNP mRNA(+) population (Supplementary Fig. S10). These reporter false negatives have been previously reported^26^, and in our context help to explain why we only observed relatively mild downward fold-changes up to a factor of 2, measured by RNA-seq, for pluripotency genes in our Nanog:VNP(−) population. Nonetheless, VNP transcripts were at least ten times less abundant in Nanog:VNP(−) than in Nanog:VNP(+) in our RNA-seq experiments, confirming both the purity of our sort and the correlation between VNP protein and RNA levels.

Our RNA-seq data revealed upwards fold-changes of lineage-associated genes in the Nanog:VNP(−) subpopulation mostly between factors of 4 and 8. These expression differences, however, may either be uniformly present across all Nanog:VNP(−) cells, or may be confined to sub-subpopulations within the Nanog:VNP(−) fraction. In particular, we had two questions: i) how widely and to what extent within the Nanog:VNP(−) are lineage markers expressed; i.e., are they limited to a fraction of *Nanog* mRNA(−) cells that have likely departed from pluripotency? and ii) does lineage-associated gene expression arise in a coherent fashion, with strong overlap of markers for a particular lineage, indicating relatively advanced stages of lineage commitment? We used single cell RNA FISH on a large set of cells to answer these questions.

To address the first question, we assessed the uniformity of lineage marker expression within the *Nanog* mRNA (−) subpopulation by counting *Nanog*, *Crabp2* (ectoderm lineage) and *T* (Brachyury, mesendoderm lineage) transcripts in Nd cells (*N = *2360) grown in 2i + LIF conditions (Fig. 4B). We found that only a small percentage of cells (18%, *N *= 138) within the set of *Nanog* mRNA (−) cells express any of the 2 examined lineage-specific markers; however, when they do express them, it is at the level of tens (Crabp2) or hundreds (T) of transcripts (Fig. 4B and Supplementary Fig. S9). The majority of cells expressing *T* and the vast majority expressing *Crabp2* have low levels of Nanog mRNA. Also, even those cells that were positive for both *T/Crapb2* and *Nanog* still displayed much lower levels of Nanog than the vast majority of the *Nanog*(+) subpopulation. We concluded that the expression of lineage markers is generally confined to a small subset of the *Nanog* mRNA (−) cells, and is not broadly present in the Nanog:VNP(−) subpopulation. Interestingly, we observed 2/2360 cells that simultaneously expressed both of these lineage markers, which in normal development are expected to be expressed in mutually exclusive sets of cells.

We further investigated the maturity of the lineage specification programs that are active in cells with low levels of *Nanog* mRNA by looking for the coexpression of genes belonging to overlapping lineages. Using the specific example of the mesodermal markers *T* and *Tbx6*^27^, we observed that, out of 764 cells, only two expressed *Tbx6* mRNA and these were a subset of the seven *T* mRNA expressing cells (Fig. 4C). We note that *T* and Tbx6 are independent mesoderm markers in the sense that the onset of *Tbx6* expression does not require the activity of *T*^27^, suggesting that a full-scale mesodermal program can be underway in a very small fraction of cells despite the continued presence of 2i and LIF in the culture.

## Discussion

The heterogeneous character of an ESC population has been the subject of intense study in recent years^3^,^4^,^5^. One of the central reasons for studying stem cell expression heterogeneity is the notion of reversible lineage priming, in which some cells will spontaneously and transiently deviate from a purely pluripotent expression profile, and consequently, upon exposure to differentiation cues will be primed to differentiate. An alternative hypothesis would be that the stem cell state is akin to an unstable equilibrium in which cells that fluctuate away from pluripotency are unable to return to pluripotency^35^.

The most direct way to distinguish between these alternatives is to visualize (via time-lapse imaging of a fluorescent protein marker) whether a cell whose expression of a given marker dips will eventually recover expression, which is something that several studies have shown^6^,^7^,^10^,^11^,^16^. These studies, however, typically suffer from reporter false-negative issues, a milder but still present issue in our reporter cell line, in which a cell may exhibit low levels of a fluorescent marker, but high levels of the relevant mRNA. It is thus unclear whether all cells observed as “returning” were ever really negative for the relevant pluripotency gene to begin with. For instance, many of these studies appear to demonstrate “return” by culturing subpopulations of either marker-high or marker-low cells isolated by fluorescence-assisted cell sorting and showing that both the high and low populations will ultimately regenerate the original population distribution. However, a key problem with this interpretation is that the rate of the marker-high population regenerating the entire population is typically much faster than the rate of the marker-low population doing the same thing. A mathematical analysis (Supplementary Material section 2) shows that the rates of population regeneration should be the same in both directions. Further, the fact that the putatively marker-negative population takes much longer to recover can be explained by a model (Supplementary Material sections 1,3; Supplementary Fig. S11, S12) in which this population consists of some cells that are actually Nanog-negative and do not return to the Nanog-positive state while the rest of the cells are actually Nanog-positive and thus can rapidly return to a marker-positive state; i.e., the alternative “unstable equilibrium” hypothesis. This does not preclude the possibility that some cells undergo reversible lineage priming, but establishes the plausibility of the alternative.

It is on the basis of these results that we analysed in detail the gene expression profile of marker-negative cells to try to understand their different states. In our experiments, we took ESCs growing in serum+LIF and transferred them to 2i + LIF conditions for 2 days, after which we (1) sorted the Nanog:VNP(+) and Nanog:VNP(−) cells for RNA-sequencing and (2) performed RNA FISH analysis. The observed transcriptome signature of the Nanog:VNP(−) cells suggested that this subpopulation of cells includes a substantial fraction that express lineage markers and had departed from the pluripotent state. Our analysis shows that this fraction comprises around two thirds of the Nanog(−) cells and do not exhibit transient and shallow departures from pluripotency; indeed, they show many signs of being further from pluripotency than cells subjected to 1 day of directed differentiation. Our transcription factor binding site analysis showed that, to a great extent, the genes that are up-regulated in Nanog:VNP(−) cells are not under the proximal control of the pluripotency network. Also, the fold-reduction of many pluripotency genes in Nanog:VNP(−) is larger than would be expected for relatively short-term fluctuations (on the order of several hours). As a specific illustration, the very low levels of *Nanog* and *VNP* RNA determined by RNA FISH in the Nanog(−) population (Supplementary Fig. S9 and S10) suggest that most Nanog mRNA(−) cells have stopped transcribing Nanog for a time equivalent to several *Nanog* RNA lifetimes.

Given our experimental design, there are two possible interpretations of these results in light of the reversible lineage priming vs. spontaneous differentiation scenarios. One is that our results provide evidence for the existence of a subpopulation of Nanog(−) cells corresponding to spontaneous differentiation that will not return to the pluripotent state, which does not exclude the possibility that there may also be a reversible lineage-primed subpopulation of Nanog(−) cells. Another is that the observed changes in gene expression are transient effects induced by transfer to 2i + LIF, most likely due to the consequent burst of WNT signaling activity caused by exposure to the GSK3-beta inhibitor, which is known to promote mesoderm differentiation in serum+LIF conditions^28^,^29^,^30^. Although our analyses indicate that ESCs have already equilibrated at the single cell level even after just 2 days in 2i + LIF conditions (Supplementary Fig. S3 and S4), it is possible that the detected spontaneously differentiating Nanog mRNA(−); Oct4 mRNA(−) cells are a consequence of this exposure to WNT signaling activity, and would explain the enrichment for mesoderm markers (T, Mixl1, Tbx6, lncRNAs) observed in the Nanog:VNP(−) transcriptome. Nevertheless, we believe our interpretation also corresponds to results documented by others in long-term 2i + LIF culture^16^.

Our single cell analysis also reveals the degree to which particular genes may plausibly be associated with reversible lineage priming. Our RNA-seq results show that Nanog:VNP(−) cells both express lower levels of pluripotency markers and higher levels of lineage markers; however, the bulk analysis cannot reveal whether these expression characteristics appear in the same cells. A priori, one possibility is that the altered expression levels of these genes could be completely uncorrelated, in which case, reversible lineage priming might plausibly occur in a cell that expresses, say, both *Nanog* and *Tbx6* simultaneously. Instead, we observed that the global changes in expression levels were relatively coherent at the single cell level. Taking *Tbx6* as a particular example, it is up-regulated in the Nanog:VNP(−) population by RNA-Seq. When we looked at *Tbx6* expression by single molecule RNA FISH, we found that spontaneously arising *Tbx6* mRNA (+) cells are also *Oct4* mRNA (−). Because Oct4 mRNA half-life is high (~7.4 hours^25^) this would imply that most *Oct4* (−) cells have not actively transcribed *Oct4* for quite a long time, a sign of permanent departure from pluripotency. This strongly indicates that Tbx6 up-regulation in the Nanog mRNA (−)/Oct4 mRNA (−) sub-population cannot be interpreted as a sign of reversible lineage priming in Nanog (−) cells, that will stochastically revert back to the pluripotent “unprimed” ground state. We speculate this might be the case for other mesoderm markers, like Mixl1 and mesogenin, which are also up-regulated in the Nanog:VNP(−) population isolated from 2i + LIF cultures. We also remark that the strong correlations between *Nanog*, *Rex1* and *Oct4* mRNA at the single cell level shows that their switching from (+) to (−) is part of a concerted change in the transcriptome profile rather than the effect of standalone transcriptional bursting^31^,^32^, potentially signifying a deviation from pluripotency.

Our single cell RNA FISH results suggest a “Russian doll” hierarchy of gene expression, in which a subset of the population is *Nanog* mRNA (−), a further subset of which is *Oct4* mRNA (−), and a further subset of which express lineage markers. Reversible lineage priming may occur at any tier of this hierarchy, although, as argued, the lack of *Oct4* mRNA expression likely indicates that some of the *Nanog* mRNA (−) cells have irreversibly left the pluripotent state. It is possible that some of these cells are in a state of transient, reversible, departure from pluripotency; for instance, we found a considerable fraction (around 30%) that are still *Oct4* mRNA (+). It is also interesting that only a small portion (less than 20%) of the *Nanog* mRNA (−) cells express any of the developmental genes *Crabp2*, *T*, and *Tbx6*. Presumably, at least some of the remaining *Nanog* mRNA (−) cells are expressing developmental genes for other pathways, or it could be that expression of these lineage markers is due to sporadic transcriptional bursts^16^. However, for the reasons we argue above, we consider it more likely that most of the gene expression changes we observed by RNA-Seq represent a deeper, irreversible departure from pluripotency.

It is interesting that spontaneously differentiated cells characterized by strong lineage-marker expression can arise even in culture conditions that strongly favor the stem cell state. Our study gives mixed results on the question of whether spontaneous differentiation leads to normal lineage progression. On the one hand we found that spontaneously-arising *Tbx6*- expressing cells also express another marker for the same lineage, *T*. However, we found just as many cells (just two), expressing both *T* and *Crabp2*, which are markers for diverging lineages. Further studies involving a more extensive array of markers and deeper sampling could help better describe the initial steps of the differentiation process in pluripotent stem cells.

## Experimental procedures

### Cell culture

In this study, we used the Nd ESC line, a BAC-transgenic line for VNP-tagged Nanog gene derived from E14tg2a ESCs^12^. E14tg2a ESCs (a kind gift from Austin Smith’s lab, University of Cambridge, UK) were used as a negative control for VNP expression. ESCs were routinely expanded in serum + LIF media (GMEM medium (Invitrogen) supplemented with ES-qualified serum (Invitrogen) and LIF), and were transferred to 2i + LIF medium (iStem medium (Stem Cells Inc.) supplemented with LIF) for 48 h prior to FACS-sorting and RNA collection. At this point, the levels of VNP had largely equilibrated (Supplementary Fig. S13), and additionally, the expression levels we measured correlated well with the RT-PCR levels of a panel of 48 genes taken from cells grown in 2i + LIF for 6 days (Supplementary Fig. S3). Additionally, ESCs were grown in 2i + LIF medium for 24 h, followed by removal of LIF and inhibitors (N2B27 medium) for another 24 h (Diff).

### Flow cytometry and sorting

Live cells flow cytometry analysis and sorting experiments were performed as described previously^12^, respectively on a FACS Calibur cytometer (Becton Dickinson) or on a FACS Aria cell sorter (Becton Dickinson). For sorting, VNP low (VNP−), VNP intermediate (VNP+) and VNP high (VNP++) Nd ESCs populations were collected and processed for RNA extraction. Bulk populations of cells grown in 2i + LIF for 48 h (Stem) or in N2B27 for 24 h (Diff) were also collected without gating for VNP levels and analysed. The whole process was repeated once to obtain a biological replicate.

### RNA extraction and sequencing

Total RNA was extracted from 10^6^ cells using a High Pure RNA Isolation kit (Roche Diagnostics) and DNAseI. We prepared libraries for RNA-sequencing by using the Illumina TruSeq kit, which includes poly-adenylation selection, following the manufacturer’s recommendation. We sequenced the libraries on an Illumina HiSeq 2000. Each sample yielded between 90 and 220 million 100 base paired-end reads.

### Differential Expression

Reads were aligned to the mouse genome (mm9 assembly) and transcriptome (obtained from the Refseq, UCSC known gene and VEGA annotations) using the RUM RNA-Seq alignment pipeline with default parameters^33^, and we found 82% to 84% of reads from each sample mapped uniquely (obtained raw counts shown in Supplementary Table S1). We used DESeq (v. 1.10.1) to test for differential expression in our RNA-Seq study^34^ (Supplementary Table S2).

We selected only one transcript model for each gene, preferring Refseq to UCSC and UCSC over VEGA, and then selecting the longest transcript. We used default DESeq size factor estimation, and estimated count variances with the “per-condition” method, “parametric” fit type, and conservatively choosing the “maximum” sharing mode. We chose not to exploit the paired structure of our experimental design to gain further statistical power. Genes were tested for differential expression between pairs of the five conditions (Nanog:VNP(−), Nanog:VNP(+), Nanog:VNP(++), Stem, and Diff) to produce log fold expression changes and p-values. Differential expression hits were obtained by controlling for the false discovery rate by the Benjamini-Hochberg procedure.

### Principal Component Analysis

Principal Component Analysis was carried out as in the DESeq vignette after re-estimating variances using the required “blind” method and applying the DESeq variance stabilizing transform.

### GO Analysis

For our “greedy” style analysis, we created a ranked list of GO categories by selecting first GO categories larger (annotated for more genes) than some size (for example 750 genes in Fig. 2), and then sequentially selecting the GO category with the highest concentration of genes in a gene subset of interest (for example, all genes that were found as hits in either Nanog:VNP(+) vs Nanog:VNP(−) or Stem vs Diff). After each step we remove all genes in the selected category and repeat until the biological process root category (GO:0008150) is selected. This procedure prevents closely related GO terms from crowding the list of GO terms. Then, given any gene, we assign it to exactly one of these selected categories, preferring the category that was selected earliest. We say that the gene “fell” into that category.

The advantages of our GO analysis over more conventional null hypothesis testing for significant enrichment of GO categories is that it provides a much simpler means to achieve the twin goals of arriving at categories of the right scope and also preventing overlapping categories, which make parsing the output difficult. There are more sophisticated methods for attaining these goals, but our method is computationally simple and straightforward. Our code can be downloaded at the following web addresses: https://gist.github.com/gauthamnair/6400111, https://gist.github.com/gauthamnair/6400293.

We also carried out more conventional null hypothesis testing for significant enrichment of GO (Biological Process) categories in gene subsets with the R package goseq^18^, using the logarithm of the average read counts of each gene in the conditions considered to account for selection bias. GO annotations were obtained from the Bioconductor package org.Mm.eg.db (v.2.7.1).

### lincRNA

Recent versions of lincRNA annotations were obtained by personal communication with Mitch Guttman and Pamela Russell. The transcripts represent updated versions of those published for mouse ESCs^22^. For each lincRNA, we used a “merged” transcript model constructed from the genomic union of its isoforms. To obtain fold changes and differential expression p-values, the entire DESeq procedure was repeated after adding these merged lincRNA and their counts to the existing gene set.

### RNA FISH

RNA FISH was carried out largely as reported previously^15^. Cells were dissociated by trypsinization, washed in PBS, fixed in 4% paraformaldehyde at room temperature and permeabilized and stored in 70% ethanol at 4 °C. All washes and hybridizations were carried out in suspension. Wash buffers included 0.1% Triton X-100 to minimize losses to sticking on the walls. Samples were mounted between coverglasses in the glucose-oxidase-based 2 × SSC anti-fade buffer described previously^15^. We imaged using a 100 × 1.4NA oil-immersion objective, a Nikon Ti-E wide field microscope, and a deep-depletion CCD camera (Pixis 1024, Princeton Instruments).

## Additional Information

**How to cite this article**: Nair, G. *et al*. Heterogeneous lineage marker expression in naive embryonic stem cells is mostly due to spontaneous differentiation. *Sci. Rep*. **5**, 13339; doi: 10.1038/srep13339 (2015).

## Supplementary Material

Supplementary Information

Supplementary Table S1

Supplementary Table S2

Supplementary Table S3
