# Supplementary material for: Heterogeneous lineage marker expression in naive embryonic stem cells is mostly due to spontaneous differentiation
Source: Sci Rep. 2015 Aug 21;5:13339. doi: 10.1038/srep13339 (PMC4544010; doi:10.1038/srep13339)
Supplement: Supplementary Information [file srep13339-s1.pdf]

Heterogeneous lineage marker expression in naive embryonic stem cells is mostly due to spontaneous differentiation

Gautham Nair[a,1], Elsa Abranches[a,2, 3], Ana M. V. Guedes[2, 3], Domingos Henrique*[,2, 3] and Arjun Raj[†1]

[1]Department of Bioengineering, University of Pennsylvania, Philadelphia, PA 19104, USA
[2]Instituto de Medicina Molecular and Instituto de Histologia e Biologia do Desenvolvimento, Faculdade de Medicina da Universidade de Lisboa, Lisboa, Portugal
[3]Champalimaud Neuroscience Programme, Champalimaud Centre for the Unknown, Doca de Pedroucos, Lisboa, Portugal

[a] Contributed equally
* henrique@fm.ul.pt,
[†] arjunraj@seas.upenn.edu

1 Discussion of population dynamics under spontaneous differentiation

Since we propose in the main manuscript that some portion of stem cell heterogeneity is best thought of as spontaneous irreversible departure from pluripotency, we address in this section how pluripotent cells can nevertheless remain a stable fraction of the population.

Suppose that a pluripotent population of ESCs (type A) has a growth rate k_A and a rate of irreversible spontaneous conversion k_{AB} into a non-pluripotent type B that grows at a rate k_B. The equations of motion for the population sizes $A(t)$ and $B(t)$ are:

$$\dot{A}(t) = k_A A - k_{AB} A \qquad \dot{B}(t) = k_B B + k_{AB} A$$

Supposing that B(0) = 0, and $A(0) = A_0$, we have:

$$A(t) = A_0 e^{(k_A - k_{AB})t}$$

And

$$\begin{aligned} e^{-k_B t} B(t) &= k_{AB} \int A_0 e^{(k_A - k_{AB} - k_B)t} \\ B(t) &= \frac{k_{AB}}{k_A - k_{AB} - k_B} A_0 \left[e^{(k_A - k_{AB})t} - e^{k_B t} \right] \end{aligned}$$

As long as the growth rate of pluripotent cells is faster than the sum of the rate of their spontaneous differentiation and the growth rate of the non-pluripotent cell population, $k_A > k_{AB} + k_B$, the ratio of differentiated cells to pluripotent cells will remain finite and equal to:

$$\frac{B(t)}{A(t)} \rightarrow \frac{k_{AB}}{k_A - k_{AB} - k_B} \qquad \text{as } t \rightarrow \infty$$

2 Discussion of rates of exchange during reversible fluctuations

In this section we show that when there are truly reversible fluctuations between two cellular states (between Nanog(-) and Nanog(+) for example), and one purifies either state, the rate at which the equilibrium is re-established is the same regardless of which state was originally purified.

The simplest model of exchange between two populations A and B (corresponding to Nanog(+) and Nanog(-)) is:

$$A \rightleftharpoons B: \quad A \overset{k_{AB}}{\rightarrow} B, \quad B \overset{k_{BA}}{\rightarrow} A$$

The equilibrium equation is:

$$\frac{\alpha^*}{\beta^*} = \frac{k_{BA}}{k_{AB}}$$

where α and β are the fraction of cells of type A and type B and * denotes the steady state value. One can show that if the equilibrium is disturbed, for example, let $\alpha = 1$ and $\beta = 0$, then the return to equilibrium follows:

$$\alpha(t) = \alpha^* + (1 - \alpha^*) e^{-(k_{AB} + k_{BA})t} \qquad \beta(t) = \beta^* \left[1 - e^{-(k_{AB} + k_{BA})t} \right]$$

The rate constant of achieving equilibrium is $k_{AB} + k_{BA}$ and is independent of whether we start with all A ($\alpha = 1$) or all B ($\beta = 1$), no matter how different the rates k_{AB} and k_{BA} might be.

For the case of pluripotency gene reporter heterogeneity, this means that marker(+) cells and marker(-) should both regenerate the full distribution in the same number of days. This is rarely seen in existing studies on stem cell heterogeneity, as depicted schematically in Figure S12. Marker(-) cell populations are much slower to regenerate marker(+).

Although at first it seems odd to expect that the usually small marker(-) subpopulations should rapidly regenerate the much larger marker(+) population, in fact, in this simple reversible scheme, the marker(-) population can only be small at steady state if it has a fast rate of return to marker(+) (see the equilibrium equation above). The alternative is that the marker(-) population is small due to a faster growth rate of (+) than (-) cells. We analyze the more complex situation in which A and B have possibly different cell cycle growth rates below, and show that after taking the effect into account, experimental curves predict extremely slow rates of return k_{BA} on the order of ten days or more.

3 General case of 2-state population recovery dynamics

A general model for exchange between populations with growth rates k_A and k_B:

$$\dot{A}(t) = k_A A - k_{AB} A + k_{BA} B$$

$$\dot{B}(t) = k_B B - k_{BA} B + k_{AB} A$$

The first section of this discussion treated the case where $k_{BA} = 0$, and the second treated the case of $k_A = k_B = 0$, which is also equivalent to the case where k_A and k_B are equal but nonzero.

First we recast in terms of the fraction of cells of each type, setting $\alpha(t) = A/A + B$ and $\beta(t) = B/A + B$:

$$\dot{\alpha}(t) = (k_A - k_B)\alpha\beta - k_{AB}\alpha + k_{BA}\beta$$

The steady state ratio is controlled by the dimensionless ratios $k_{AB}/(k_A - k_B)$ and $k_{BA}/(k_A - k_B)$ and occurs when:

$$\alpha^*(1 - \alpha^*) = \frac{k_{AB}}{k_A - k_B}\alpha^* + \frac{-k_{BA}}{k_A - k_B}(1 - \alpha^*)$$

which always has one solution $0 < \alpha < 1$ by the intermediate value theorem as long as k_{AB} and k_{BA} are nonnegative and at least one is not zero. A graphical analysis shows that in this scenario the equilibrium is stable. If the growth rates of A and B are similar, then $k_A - k_B \to 0$ and one recovers the familiar steady state result $\alpha = k_{BA}/(k_{AB} + k_{BA})$.

If one purifies A or B populations then the initial slope of the population recovery is $\dot{\alpha} = -k_{AB}$ if starting from $\alpha = 1$ and $\dot{\alpha} = k_{BA}$ if starting from $\alpha = 0$. This initial rate of recovery is independent of the growth rates of A and B.

To solve the general case, we recast the dynamics in terms of the deviation from equilibrium $\Delta = \alpha - \alpha^*$:

$$\begin{aligned}
\dot{\Delta}(t) = \dot{\alpha}(t) &= (k_A - k_B)(\alpha^* + \Delta)(\beta^* - \Delta) \\
&\quad - k_{AB}(\alpha^* + \Delta) + k_{BA}(\beta^* - \Delta) \\
&= -(k_A - k_B)\Delta^2 \\
&\quad - \Big((k_A - k_B)(\alpha^* - \beta^*) + k_{AB} + k_{BA}\Big)\Delta
\end{aligned}$$

The rate of decay of a small perturbation from equilibrium is $\kappa = (k_A - k_B)(\alpha^* - \beta^*) + k_{AB} + k_{BA}$ which returns the familiar result $\kappa = k_{AB} + k_{BA}$ for the case of no differences in growth rate. For larger deviations, the recovery from a perturbation that increases the faster growing population is faster than the recovery from a perturbation that increases the slower one:

$$\dot{\alpha}(t) = -(k_A - k_B)(\alpha - \alpha^*)^2 - \kappa(\alpha - \alpha^*)$$

The dynamics are determined by the three paramaters $k_A - k_B$, k_{BA}, and k_{AB}. These can be written in terms of the equilibrium fraction α^*, the small perturbation recovery rate κ, and the ratio of the growth rate difference to the population recovery rate $g = (k_A - k_B)/\kappa$. After some algebra:

$$\begin{aligned}
k_A - k_B &= g\kappa \\
k_{BA} &= \kappa\alpha^*(1 - g\alpha^*) \\
k_{AB} &= \kappa(1 - \alpha^*)\left[1 + g(1 - \alpha^*)\right]
\end{aligned}$$

We are interested in the case where $k_A > k_B$ since experimentally recovery from (-) is always slower than from (+). The requirement that all k be nonnegative therefore implies that $g < 1/\alpha^*$, so the range of possible values is $0 \leq g < 1/\alpha^*$. Calculated recovery curves are shown in Fig. S13.

In the plots we have labelled the implied lifetime for return from the (-) state, $1/k_{BA}$, normalized to the small-perturbation recovery lifetime $1/\kappa$. We note that changes in growth rate differential have only a minor effect on the recovery from purified (+) especially for situations in which the steady state has a high fraction of (+), as seen in most reports on pluripotency reporter fluctuations. After fixing κ and α^*, the range of k_{AB} is only $\kappa(1 - \alpha^*) < k_{AB} < \kappa(1 - \alpha^*)/\alpha^*$. On the other hand, the growth rate differential is reflected as a slowdown on the recovery from (-) and indeed the range of k_{BA} stretches all the way from $\kappa\alpha^*$ down to 0, the limit where transition to B is totally irreversible.

What is remarkable is that even a small asymmetry between the rate at which purified (-) and (+) populations return to steady state implies that at the single cell level the rate constant for a (-) cell to return to (+) is extremely small. As an example, take the right panel in Fig. S13. Supposing that the (+) population recovers to the steady state distribution on the time scale of 1 day (corresponding to $\kappa^{-1} \approx 1$day), if the (-) population recovers at the same rate (blue curve), it implies

that a (-) cell will return to (+) on a time scale of $k_{BA}^{-1} = 1.3$ days. However, if the (-) population recovers on the timescale of 3 days, say, it means that a (-) cell only returns to (+) on average after 12.6 days. If the (-) population recovers on a timescale of 4 days, the average (-) cell recovers only in 26 days. Even such mild deviations from the blue curve therefore imply that the rate of return of a (-) cell is much slower than would be expected for transient lineage priming.

To explain why dramatic reductions in k_{BA} lead only to mild slowdown in the time of population recovery from purified (-), we return to the equation:

$$\dot{\alpha}(t) = (k_A - k_B)\alpha(1-\alpha) - k_{AB}\alpha + k_{BA}(1-\alpha)$$

The last two terms describe the effect of interconversion by cells switching between (+) and (-), but the first term captures the effect of growth rate differences. If k_{BA} is very small, the initial rate of recovery from purified (-) $\alpha = 0$ is slow. But the only way for k_{BA} to be much smaller than k_{AB} while maintaining a high value of α^*, the steady state fraction of (+) cells, is if $k_A - k_B > 0$. The smaller k_{BA}, the larger the differential growth rate must be. Therefore, when k_{BA} is small, as soon as some (+) cells are produced from a (-) population, the larger growth rate of (+) cells will rapidly multiply their numbers relative to the (-) population. The return to steady state therefore becomes driven by the growth rate differential, the first term in the above equation, rather than primarily by interconversion from (-), the third term.

References

Abranches, E., Guedes, A. M. V., Moravec, M., Maamar, H., Svoboda, P., Raj, A. and Henrique, D. (2014). Stochastic NANOG fluctuations allow mouse embryonic stem cells to explore pluripotency. Development *141*, 2770–2779.

Anders, S. and Huber, W. (2010). Differential expression analysis for sequence count data. Genome Biol *11*, R106.

Guttman, M., Donaghey, J., Carey, B. W., Garber, M., Grenier, J. K., Munson, G., Young, G., Lucas, A. B., Ach, R., Bruhn, L., Yang, X., Amit, I., Meissner, A., Regev, A., Rinn, J. L., Root, D. E. and Lander, E. S. (2011). lincRNAs act in the circuitry controlling pluripotency and differentiation. Nature *477*, 295–300.

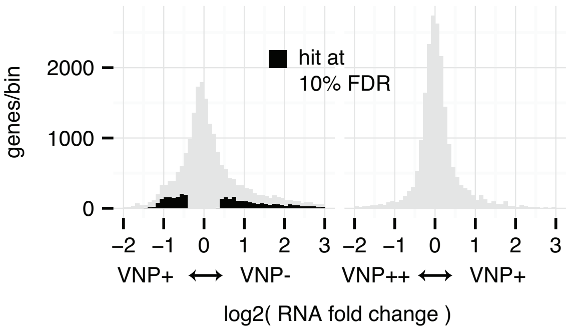

Figure S1: **VNP++ and VNP+ are Similar**. Histogram for the log fold changes of expression by RNA-Seq for 25313 genes between VNP+ and VNP- samples (left) and between VNP++ and VNP+ samples. Only 6 genes are 10% FDR hits for differential expression between VNP+ and VNP++, nearly three orders of magnitude less than between VNP+ and VNP-.

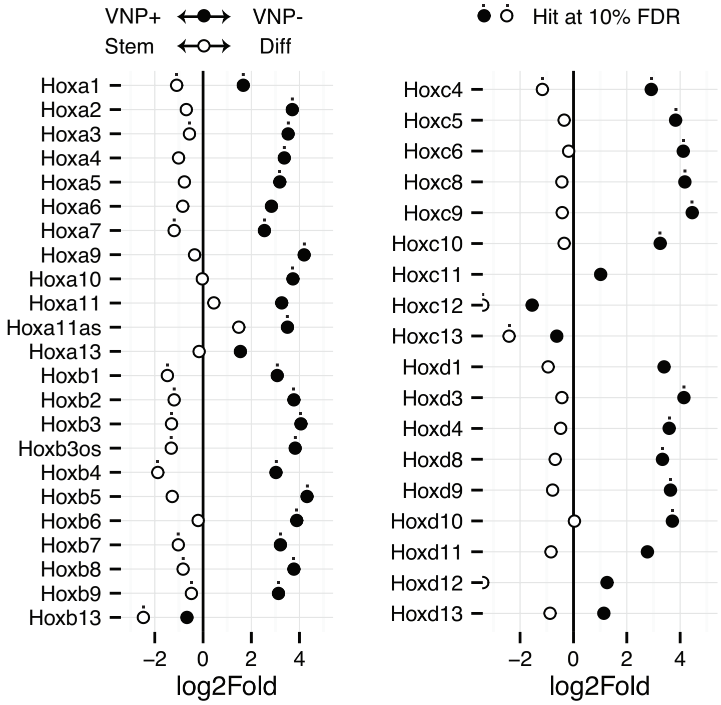

Figure S2: **Differential Expression of *Hox* genes** Fold changes of *Hox* genes in the VNP(+)/VNP(-) and Stem/Diff comparisons. The *Hox* genes are more highly expressed in *Nanog*:VNP(-) than *Nanog*:VNP(+), but lower in Diff than in Stem.

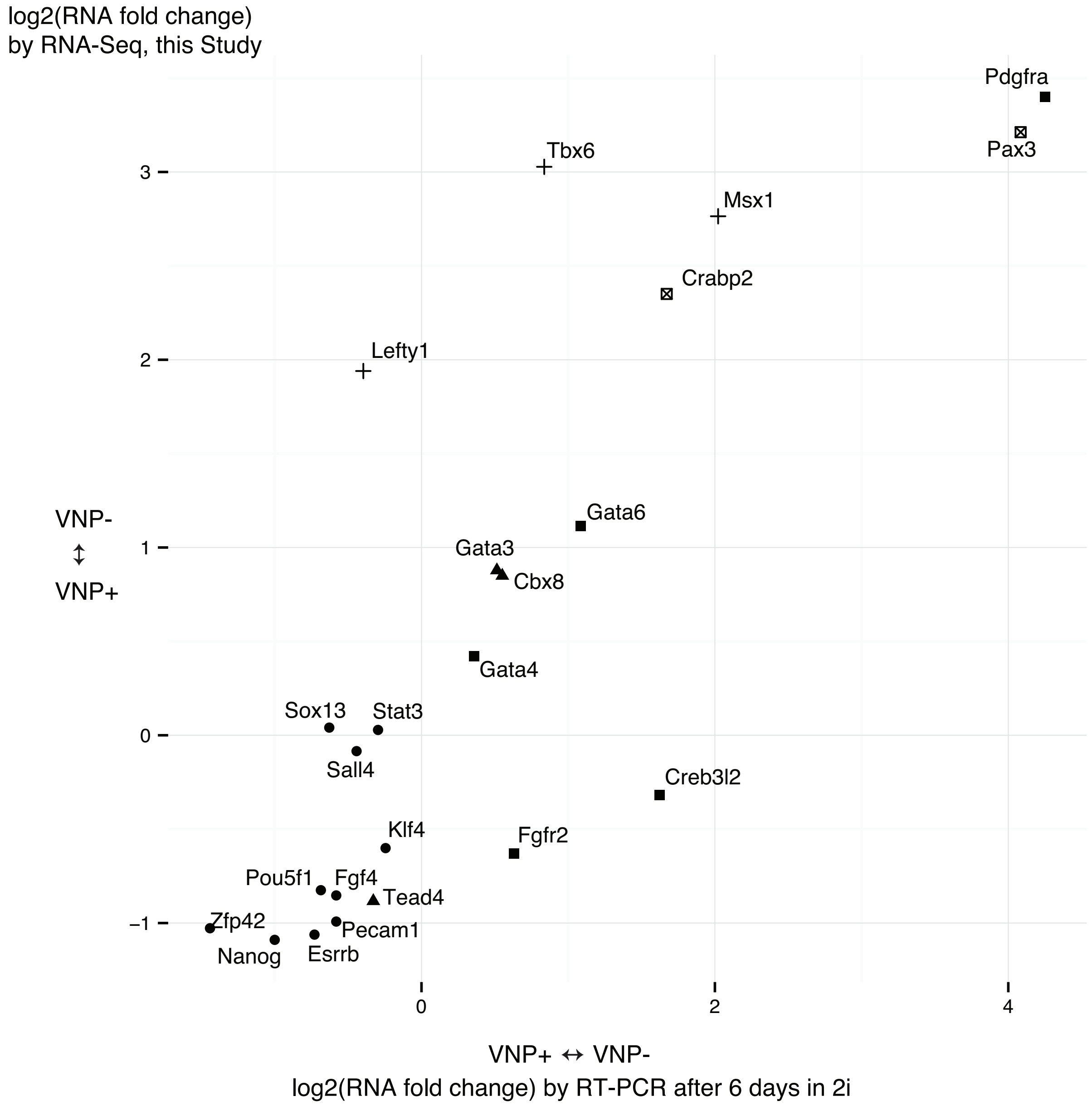

Figure S3: **Comparison of RNA-Seq and RT-PCR** Comparison of RNA-Seq fold-changes from this study between *Nanog*:VNP(+) and *Nanog*:VNP(-) sorted samples and reported fold-changes Abranches et al. (2014) measured by RT-PCR for the same cell line sorted into *Nanog*:VNP(+) and *Nanog*:VNP(-) after 6 days of culture in 2i/LIF.

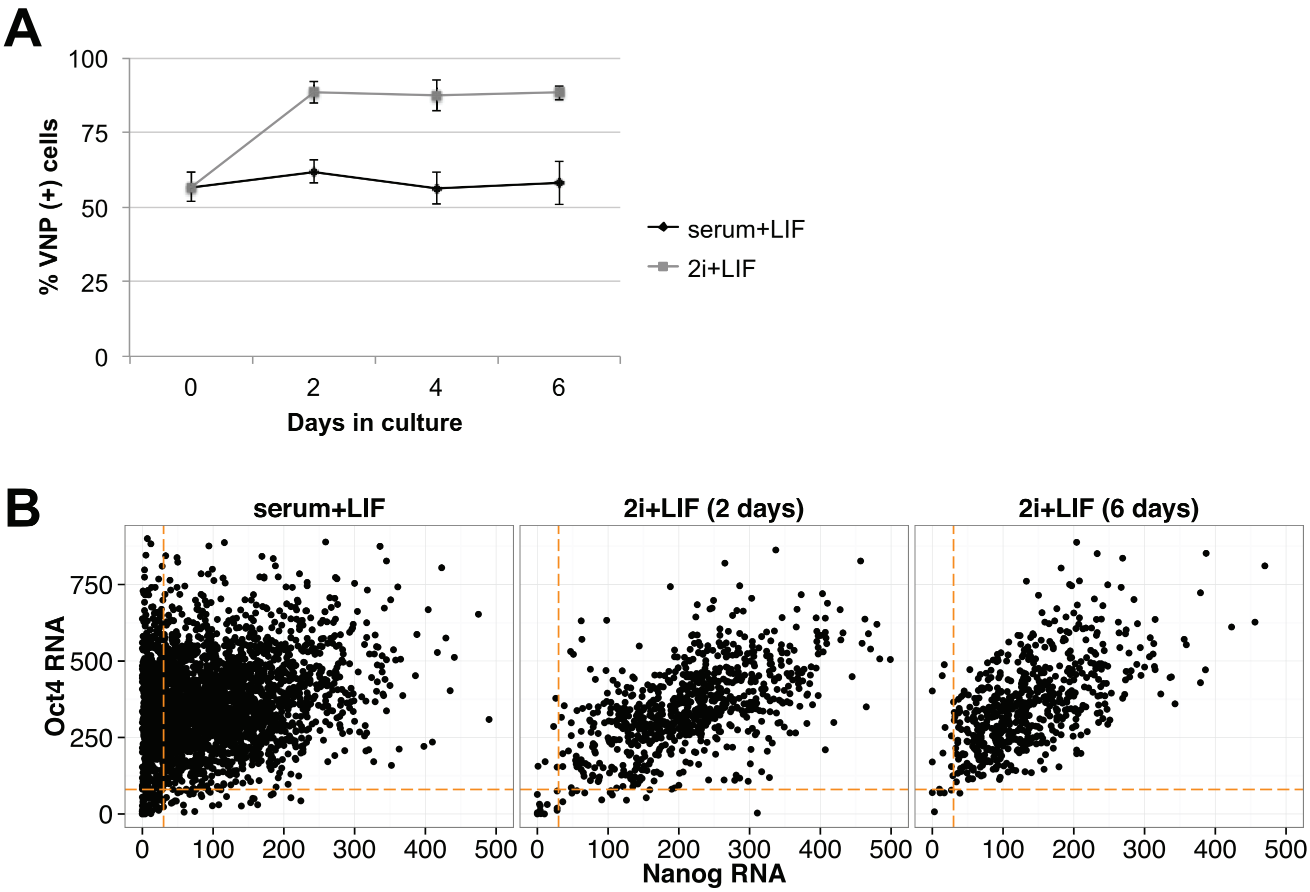

Figure S4: **Analysis of population heterogeneity after prolonged culture in 2i+LIF conditions** (Serum+LIF conditions are presented as control). (A) Fraction of VNP(+) cells determined by flow cytometry following transfer of cells from serum+LIF to 2i+LIF. Cells were monitored during 6 days showing no further changes after 2 days. (B) RNA counts determined by single-molecule RNA-FISH for *Nanog* and *Oct4* from a measurement on 3040, 782 and 618 Nd ESCs grown in serum+LIF, 2i+LIF for 2 days and 2i+LIF for 6 days, respectively. The cutoff for Nanog(+/-) and Oct4(+/-) is shown as a dashed line on the plot. Note that the % of Nanog(-) cells relative to the total number of cells changes from 22% to 2.3% upon serum+LIF to 2i+LIF transition in 2 days but then is kept constant until day 6.

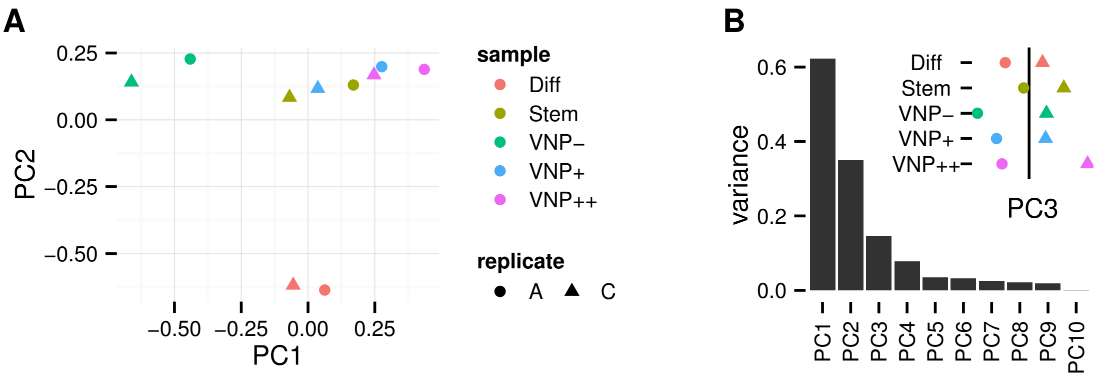

Figure S5: **RNA-Seq Principal Components Analysis**. Principal component analysis was performed after variance stabilization using DESeq Anders and Huber (2010). (A) Weights of the 10 samples in the first two principal components of our RNA-Seq experiment. Colors denote different conditions, and the two symbols are the two biological replicates. (B) Relative variance of all principal components. Inset shows the weights of the third principal component using the same symbology as in A, showing that it captures gene expression changes between replicates. Together, the plots show that VNP-correlated heterogeneity and differentiation account for most of the sample-to-sample variation of gene RNA levels.

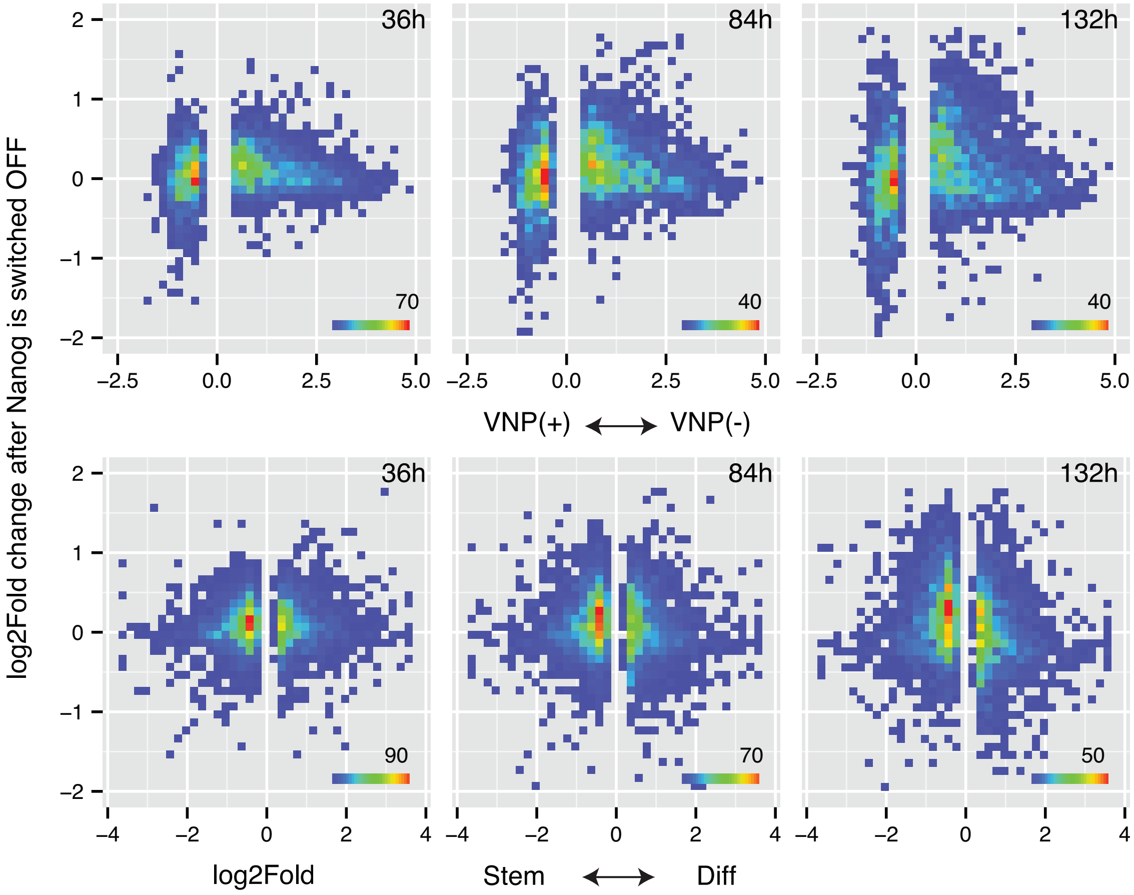

Figure S6: **Comparison to Nanog Knockdown Time Course** (top) Joint frequency of fold changes observed by microarray by MacArthur and Sevilla et al. at specified time points after external down-regulation of *Nanog*, against our RNA-Seq fold changes between VNP(+) and VNP(-) for genes that are 10% FDR hits in the VNP(+)/VNP(-) comparison. For the literature data, we used the average of all replicates and all probes corresponding to each gene. (bottom) Same but showing only 10% hits and fold changes between Stem and Diff conditions.

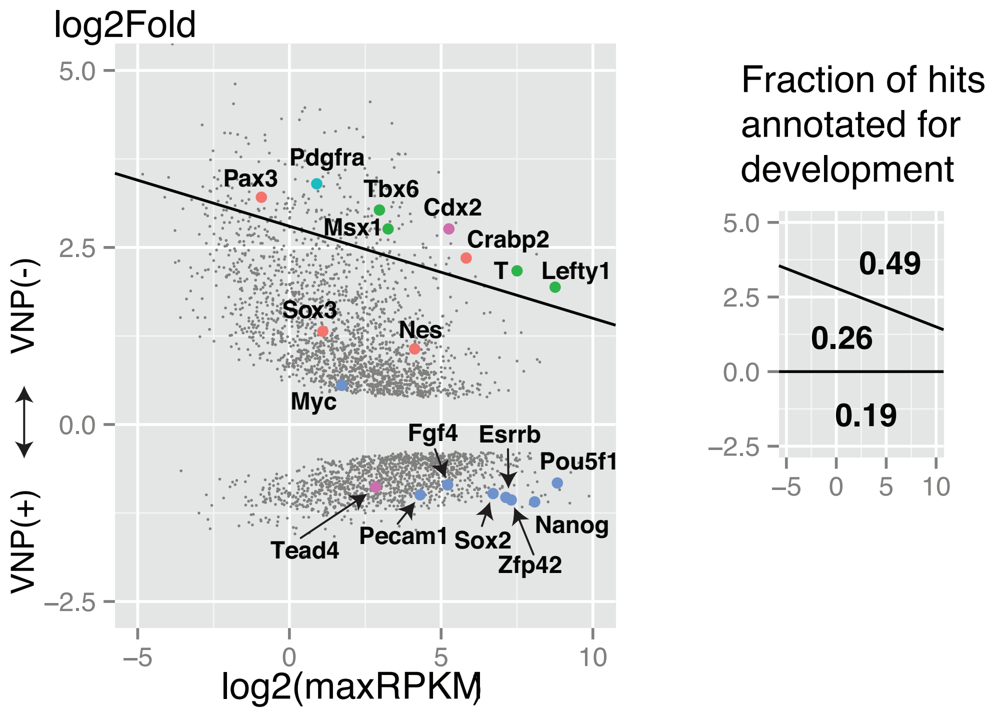

Figure S7: **The VNP(-) Transcriptome is Distinguished by Lineage-Associated Genes** (Left) Every point represents a gene that is a 10% FDR differentially expressed hit between *Nanog*:VNP(+) and *Nanog*:VNP(-). The y axis is the log fold change from VNP+ to VNP(-) and the x-axis is the log RPKM in VNP(+) for genes higher in VNP(+) and in VNP(-) for genes higher in VNP(-). Selected genes are denoted with large symbols, and have the same color if they are markers for the same cell lineages or tissues. The black line is a guide for the eye that separates the more extreme genes from the rest of the population. (Right) Fraction of the hits in each sector of the plot on the left that are annotated for at least one of embryo development (GO:0009790), anatomical structure development (GO:0048856), or pattern specification process (GO:0007389). The most salient hits in VNP(-) are highly enriched for these categories. Categories: Pluripotency (blue); Trophectoderm (pink); Mesoderm (green); Primitive endoderm (light blue); Ectoderm (red).

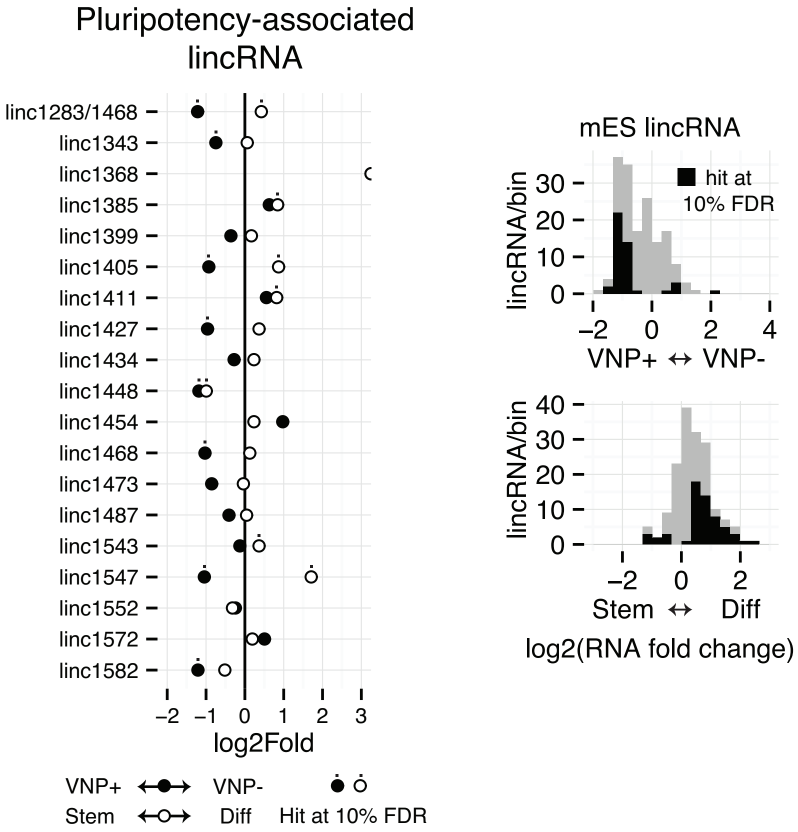

Figure S8: **Differential Expression of certain lincRNA** (Left) Fold changes of lincRNA genes identified by Guttman et al. (2011) as associated with the pluripotency network. (Right) Fold change distributions and hit status for all ESC lincRNAs (Guttman et al. (2011) and Guttman, personal communication).

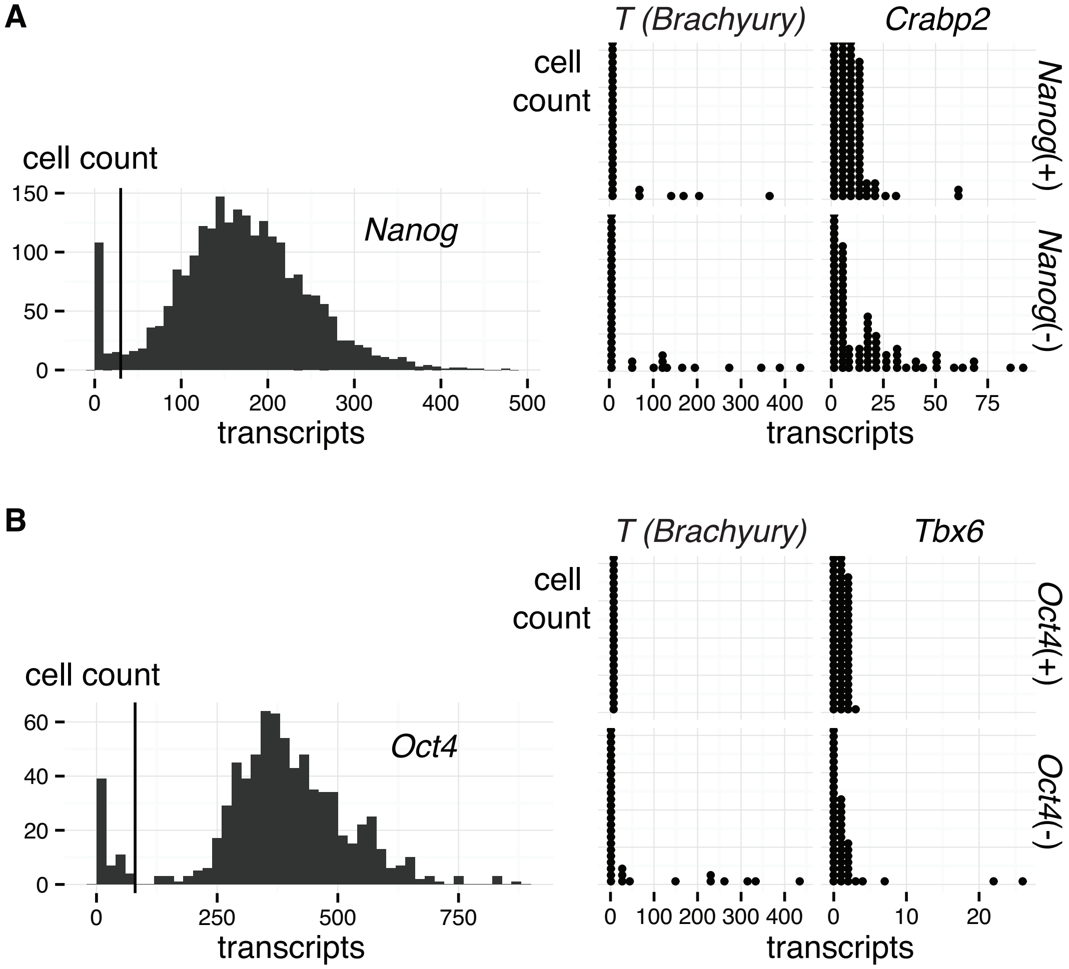

Figure S9: **Experimental transcript number distributions of *Nanog*, *T*, *Crabp2*, *Oct4*, and *Tbx6*** (A) Transcript number distributions for *Nanog*, *T*, and *Crabp2* probed simultaneously. The cutoff for *Nanog*(+/-) is shown as a straight vertical line on the plot at the left, and the *T* and *Tbx6* distributions are shown separately for *Nanog*(+) and *Nanog*(-) cells. (B) Similar to A for *Oct4*, *T*, and *Tbx6* probed simultaneously.

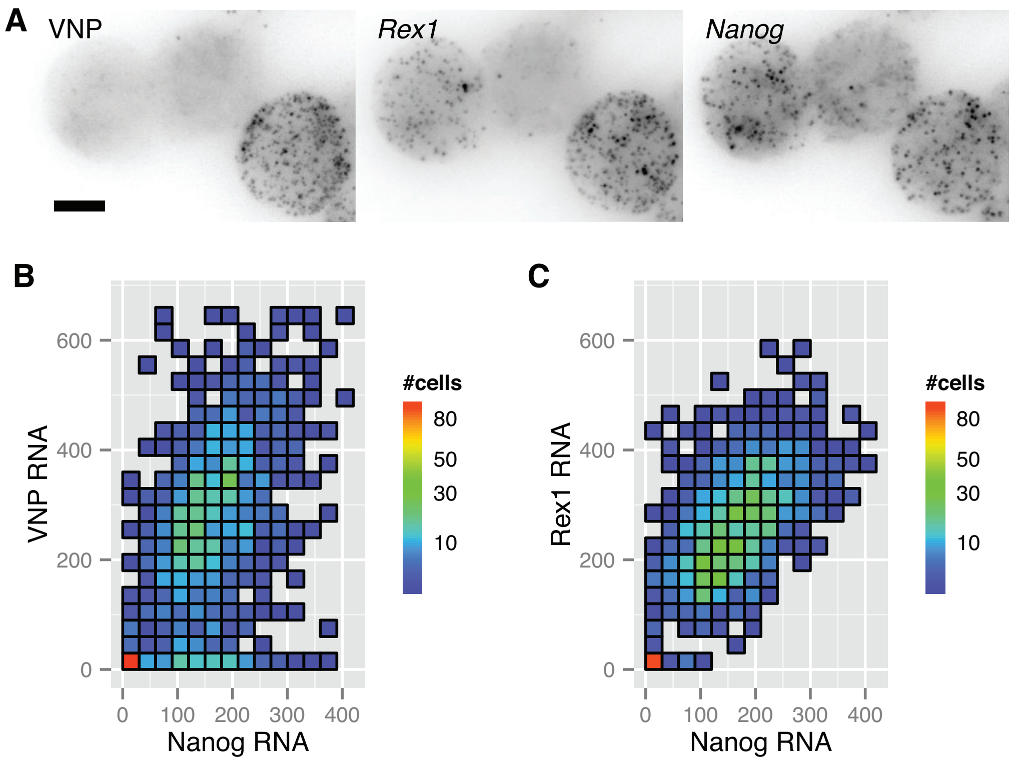

Figure S10: **Single Cell Analysis of *Nanog*, *Rex1*, and VNP reporter correlation** (A) Maximum projection of images from Nd cells stained for *Nanog*, *Rex1*, and Venus fluorescent protein (VNP). Scale bar is 5 μm. The left-most cell is an example of a VNP reporter false negative. (B) Joint frequency of RNA counts determined by single molecule RNA-FISH for *Nanog* and Venus (VNP) from a measurement on 1177 Nd ESCs grown in 2i+LIF. Note a fraction of cells with low VNP RNA counts but normal *Nanog* RNA counts. (C) Joint frequency of *Nanog* and *Zfp42* (*Rex1*) RNA from the same set of cells. These RNA levels are highly correlated.

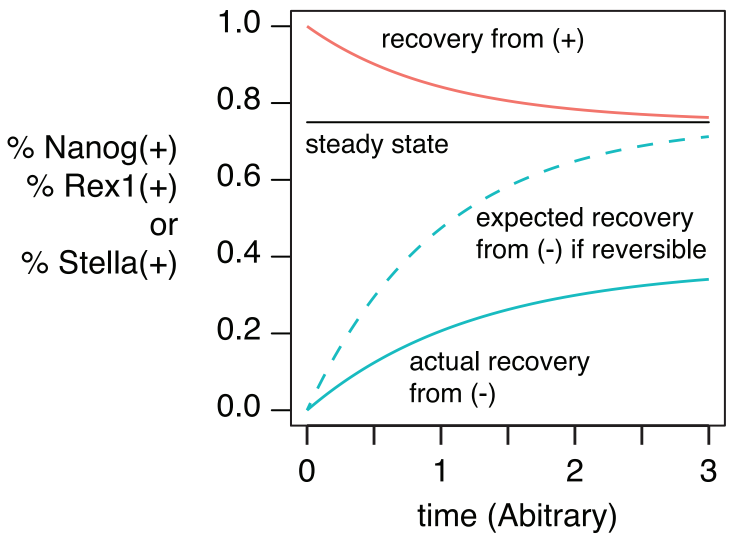

Figure S11: **Calculated curves for a model of population recovery with no growth differences**. The recovery from (+) and expected recovery from (-) are calculated assuming that they are part of a completely reversible equilibrium of cells that grow at the same rate. In experiments, the actual recovery from (-) is found much slower than expected given the recovery from (+).

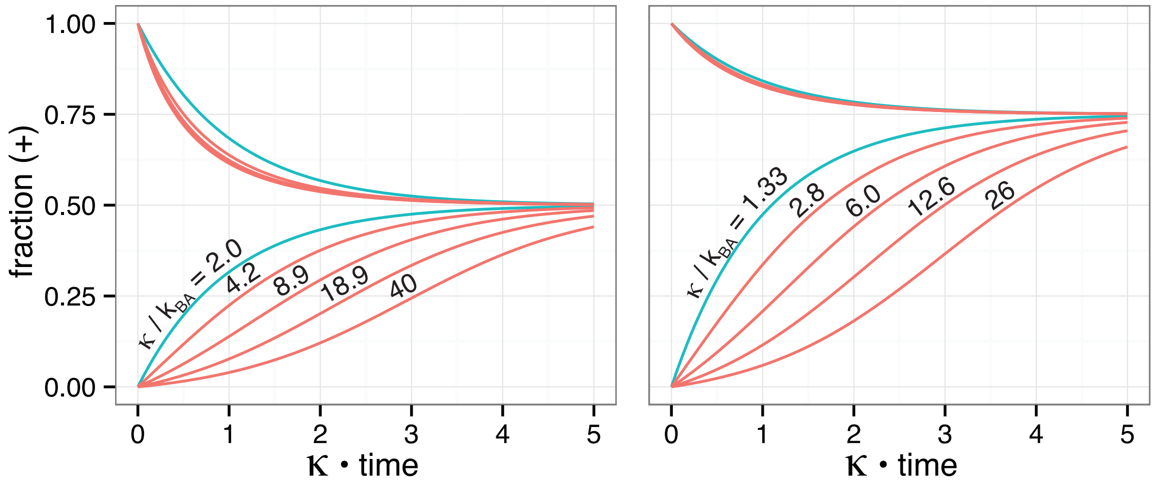

Figure S12: **Calculated population recovery curves allowing for growth rate differences.** κ is the rate constant for recovery from a small perturbation from steady state, and k_{BA} is the rate constant for individual (-) cells switching to (+). The left and right panels are calculations for steady state (+) fractions of 0.5 and 0.75 respectively ($\alpha^* = 0.5$ and 0.75). The blue line corresponds to no growth rate difference $k_A = k_B$, and the red lines show the effect of increased growth rate of population A with respect to B. See supplementary discussion for a more thorough explanation.

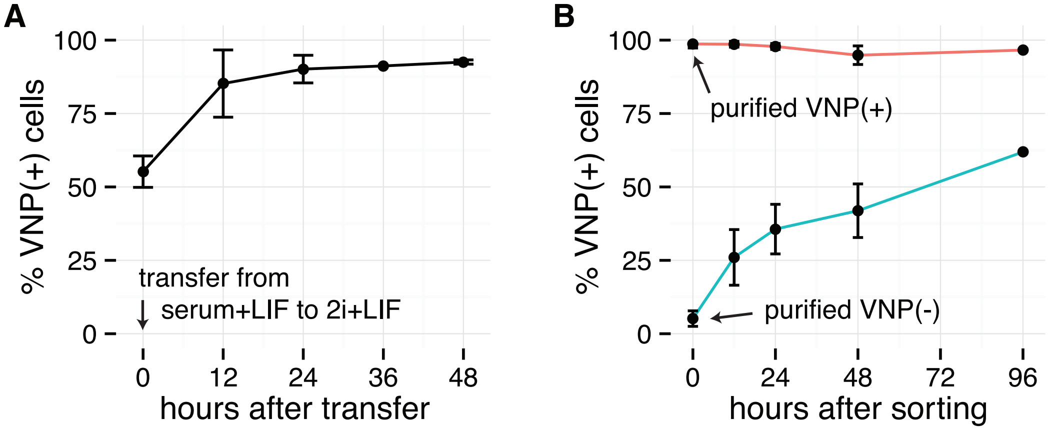

Figure S13: **Population relaxation of VNP(+) fraction** Fraction of VNP(+) cells determined by flow cytometry following (A) transfer of cells from serum+LIF to 2i+LIF or (B) plating purified VNP(+) and VNP(-) populations grown in 2i+LIF.
